# Differences in systemic inflammation and prethrombotic biomarkers between AECOPD patients with and without pulmonary hypertension

**DOI:** 10.1186/s12950-026-00508-7

**Published:** 2026-07-15

**Authors:** Xiao Bo Wang, Zhu Qi Gao, Ying Yu, Yang Fei Lu

**Affiliations:** 1 Fuyang First People’s Hospital in Hangzhou, Hangzhou, 311400 China; 2 School of Pharmaceutical Sciences, Wenzhou Medical University, WenZhou, 325035 China; 3 The First Affiliated Hospital of Wenzhou Medical University, WenZhou, 325035 China; 4 Respiratory Medicine, Fuyang First People’s Hospital in Hangzhou, Hangzhou, China

**Keywords:** AECOPD, PH, Inflammatory cytokines, Prothrombotic markers

## Abstract

**Objective:**

The relationship between inflammatory cytokines and prothrombotic markers with PH during the acute phase of acute exacerbation of chronic obstructive pulmonary disease (AECOPD) remains unclear. This study aims to compare the profiles of peripheral blood biomarkers in AECOPD patients with and without Pulmonary Hypertension(PH), and to evaluate their diagnostic significance.

**Methods:**

We conducted a case-control study enrolling 148 patients admitted to the Department of Respiratory Medicine at Fuyang First People’s Hospital in Hangzhou, Zhejiang Province, China, between January 2023 and April 2025. The patients were categorized into three groups: a control group (*n* = 49), an AECOPD group (*n* = 59), and an AECOPD with PH group (*n* = 40). The clinical characteristics and the relationships between inflammatory cytokines and prothrombotic markers were compared among the three groups. Logistic regression analysis was employed to identify inflammatory biomarkers and prothrombotic markers with independent predictive value. The predictive accuracy of these risk factors was assessed using receiver operating characteristic (ROC) curves.

**Results:**

Compared with healthy volunteers, AECOPD patients showed elevated levels of PIV, SII, IL-8, CRP, NLR, and PLR (*p* < 0.0001) based on quartile comparisons. Levels of these inflammatory markers were significantly elevated in both AECOPD and AECOPD + PH groups compared to controls (*p* < 0.0001). However, no significant differences were found between the AECOPD and AECOPD + PH groups. Among the prethrombotic markers, D-dimer and TAT were elevated in AECOPD patients with PH. ROC curve analysis identified PIV, SII, IL-8, CRP, NLR, and PLR as significant predictors of AECOPD. Among the inflammatory markers, SII exhibited the highest area under the curve (AUC) of 0.834 (95% CI: 0.748–0.905). The combination of SII and IL-8 yielded the highest AUC among inflammatory markers, at 0.926 (95% CI: 0.876–0.967). Furthermore, the combination of D‑dimer and TAT resulted in a higher AUC than either marker alone, with an AUC of 0.919 (95% CI: 0.865–0.966) for identifying PH in AECOPD patients.

**Conclusion:**

AECOPD patients with PH exhibit a distinct biomarker pattern characterized by a plateau in systemic inflammation alongside progressive coagulation activation. We speculate that this may be attributed to a regionalized redistribution of the inflammatory response or functional exhaustion of the immune system. The combination of inflammatory markers (SII and IL-8) and prothrombotic markers (D-dimer and TAT) provides excellent discriminative ability for diagnosing AECOPD and identifying concomitant PH, respectively. These findings support the use of combined biomarker panels for individualized risk assessment and early detection of PH in AECOPD patients.

**Clinical trial registration number:**

Not suitable.

**Supplementary Information:**

The online version contains supplementary material available at https://doi.org/10.1186/s12950-026-00508-7.

## Introduction

Chronic obstructive pulmonary disease (COPD) is the third leading cause of death worldwide [1] and the fifth leading cause of death in China [2]. It is characterized by high morbidity and mortality rates [3, 4]. An acute exacerbation of COPD (AECOPD) refers to an acute worsening of the condition [5], marked by the deterioration of one or more symptoms such as increased cough, worsened dyspnea, increased sputum production, and changes in sputum consistency [3]. Importantly, AECOPD is a major cause of hospitalization and mortality among COPD patients, with in-hospital, 3-month, 6-month, and 2-year mortality rates of 6.7%,18%,26%,and 31% [6–8], respectively. Pulmonary hypertension (PH) is a common and serious complication of COPD [6] and is closely associated with poor prognosis [9]. Studies have shown that the mortality rate among AECOPD patients with PH is significantly higher than that among those without PH, with a 28-day mortality rate ranging from 15.73% to 20.83% in patients with PH [9], while the mortality rate in the non-PH group is significantly lower. Furthermore, higher grades of PH are associated with increased mortality [10].

The coexistence of COPD and PH exists within a state of heightened systemic inflammation [11, 12], which promotes the formation of venous thrombosis. This aberration in coagulation function is particularly pronounced during episodes of AECOPD [13]. A hypercoagulable state is also termed a prothrombotic state (PTS) [14, 15]. It represents a pathological process involving an imbalance of the haemostatic, coagulation, and anticoagulation systems caused by multiple factors [16]. The resultant alterations within the haematological system predispose individuals to thrombus formation [17]. 

Research indicates that abnormal coagulation status and thrombosis can accelerate the progression of COPD and hasten the development of pulmonary hypertension [18]. Thrombosis increases pulmonary vascular resistance, consequently leading to a remarkably high probability (up to 92.6%) of cor pulmonale in AECOPD patients. Studies have reported that the combination of inflammatory indices such as neutrophil to lymphocyte ratio (NLR), platelet to lymphocyte ratio (PLR), and systemic immune/inflammatory index (SII) [19] provides stronger predictive power and clinical evidence than any single marker alone. Significant correlations have been observed between NLR, PLR, SII and traditional markers like C-reactive protein (CRP)and procalcitonin (PCT) in AECOPD patients, with elevations of these markers being highly significant [20]. In both AECOPD and AECOPD-PH, the concurrent elevation of NLR/PLR/SII with CRP/PCT often indicates exacerbated systemic inflammation and immune dysregulation [21]. Numerous studies have demonstrated that combined use of these markers (e.g., high NLR + high CRP, high SII + high PCT) predicts disease severity, treatment response, complications (such as respiratory failure), length of hospital stay, readmission rate, and mortality more accurately than individual parameters. These biomarker combinations may serve as independent risk indicators for both AECOPD and AECOPD-PH patients.

Under physiological conditions, severe inflammation activates the coagulation system and suppresses the fibrinolytic system [22], while simultaneously causing injury to vascular endothelial cells.D-dimer, TAT (thrombin-antithrombin complex), PIC (plasmin-α2-plasmin inhibitor complex), and t-PAIC (tissue plasminogen activator-inhibitor complex) [23] serve as standard biomarkers in AECOPD for assessing VTE [24] risk, disease severity, and prognosis. They are also key indicators of chronic hypercoagulability and microthrombosis prognosis in patients with stable COPD and PH [25]. 

It remains unclear whether the presence of PH in AECOPD [26] constitutes a straightforward amplification of systemic inflammation and hypercoagulability, or represents a distinct pathophysiological phenotype with a divergent biomarker signature.Therefore, this study was designed to conduct a cross-sectional comparison across three participant groups to delineate the differences in inflammatory and prothrombotic biomarkers and to evaluate their potential diagnostic value for AECOPD complicated by PH.

## Materials and methods

### Study design

This case-control study was conducted at the First People’s Hospital of Fuyang District, Hangzhou, China. Consecutive patients admitted to the Department of Respiratory Medicine between January 2023 and April 2025 were enrolled as the case group, while age- and sex-matched healthy controls were concurrently recruited during the same period. All participants provided written informed consent prior to study inclusion. The study protocol received ethical approval from the Institutional Review Board (IRB) of the First People’s Hospital of Fuyang District in accordance with the Declaration of Helsinki.

### Study population

Consecutive patients admitted to the Department of Respiratory Medicine were screened for eligibility. The inclusion criteria were as follows:

1. Age and Disease Stability: Patients aged 55 to 80 years with a physician-diagnosed COPD history of at least 5 years were included. This age range was selected to focus on the predominant COPD demographic while minimizing the confounding effects of extreme age-related comorbidities. Although the typical age range for COPD screening is 40–80 years [27], epidemiological evidence indicates that COPD prevalence increases substantially with advancing age, with a marked rise among those aged 55 years and older globally [28]. Furthermore, studies comparing spirometric diagnostic criteria (GOLD, NICE, and LLN) have demonstrated that the concordance between these methods progressively declines with age, with a notable decrease in agreement after age 55, supporting a narrowed lower age boundary to reduce diagnostic heterogeneity [29]. Domestically, the COPD screening questionnaire (COPD-SQ) used in Chinese clinical practice assigns progressively higher risk scores to individuals aged 50 years and above, reinforcing the rationale for focusing on this older demographic [30]. The minimum 5-year disease history was required to ensure the inclusion of patients with established, stable disease phenotypes. Evidence from longitudinal cohort studies has shown that spirometrically-defined COPD states may be unstable in the early course of disease, with transitions between diagnostic categories occurring over time; a follow-up duration of at least five years is necessary to achieve a more stable disease classification [31]. The upper age limit of 80 years was set to exclude patients with extreme age-related comorbidities that could confound outcome assessment.
2. AECOPD Diagnosis: All patients were admitted for an Acute Exacerbation of COPD (AECOPD), defined and confirmed according to the Global Initiative for Chronic Obstructive Lung Disease (GOLD) 2022 report. An AECOPD was characterized by an acute worsening of respiratory symptoms (increased dyspnea, cough, sputum volume, and/or purulence) beyond normal day-to-day variations, leading to a decision to initiate hospital management.
3. PH Diagnosis and Grouping: Patients were stratified into the PH subgroup based on transthoracic echocardiography (TTE) performed within 48 h of admission. In accordance with the 2022 ESC/ERS Guidelines and common practice in COPD-PH research, an estimated resting pulmonary artery systolic pressure (PASP) of ≥ 40 mmHg was used as the threshold indicating a high probability of PH, defining the AECOPD + PH group. Patients with a PASP < 40 mmHg were accordingly allocated to the AECOPD-only group. We acknowledge that right heart catheterization (RHC) remains the diagnostic gold standard for PH. TTE was employed here as a pragmatic, non-invasive screening tool within the clinical context of acute hospitalization; this methodological approach, while standard for such studies, may introduce classification bias compared to RHC and underscores the need for future validation in cohorts defined by invasive hemodynamics.
4. Control Group: Age-and sex-matched healthy volunteers with no history of chronic cardiopulmonary disease, normal spirometry, and no acute illness were recruited as controls.

Exclusion Criteria (applied to all participants):

- Other known causes of PH (e.g., left heart disease, chronic thromboembolic PH, connective tissue disease). 2. Active malignancy or other systemic inflammatory/autoimmune diseases. 3. Severe hepatic or renal dysfunction. 4. Current use of therapeutic anticoagulation. 5. History of venous thromboembolism within the past 6 months. 6. Inability or refusal to provide written informed consent.

### Data collection, variables, and blood sampling

#### Clinical and demographic data collection

Baseline demographic and clinical characteristics were systematically obtained from electronic medical records and supplemented by standardized patient interviews upon admission. The collected variables included age, sex, body mass index (BMI), smoking history, history of hypertension, and the length of hospital stay (also presented as “Hospitalization days” in tables).

#### Definition of key variables

##### Smoking history

Smoking status was meticulously classified and quantified due to its central role in COPD. Assessment followed a structured protocol:

##### Status classification

Patients were categorized as **current smokers** (active smoking or cessation within the past 6 months), **former smokers** (cessation > 6 months prior), or **never smokers** (lifetime exposure of < 100 cigarettes).

##### Quantification of exposure

For current and former smokers, the average number of cigarettes smoked per day and the total duration of smoking in years were recorded. Cumulative tobacco exposure was calculated and expressed as pack-years using the standard formula: Pack-years = (cigarettes per day / 20) × years smoked.

##### History of hypertension

This was defined as a binary variable (yes/no) based on any of the following: (1) a pre-existing diagnosis documented in the medical record, (2) current use of antihypertensive medication, or (3) repeated systolic/diastolic blood pressure measurements ≥ 140/90 mmHg during the current hospitalization.

#### Timing of blood sample collection

To accurately capture biomarker profiles during the acute exacerbation phase, peripheral venous blood samples were collected in EDTA and sodium citrate tubes within 24 h of hospital admission, prior to the administration of systemic corticosteroids or anticoagulant therapy.

#### Laboratory assays

Processed plasma and serum samples were analyzed for the following parameters using standardized automated platforms and commercial assay kits:

- Complete Blood Count: To derive absolute counts of neutrophils, lymphocytes, monocytes, and platelets.
- Inflammatory Biomarkers: High-sensitivity C-reactive protein (CRP) and Interleukin-8 (IL-8).
- Coagulation and Fibrinolysis Biomarkers: D-dimer, thrombin–antithrombin complex (TAT), plasmin-α2-plasmin inhibitor complex(PIC), and tissue plasminogen activator–inhibitor complex (t-PAIC).
- Derived Inflammatory Indices: The following indices were calculated:
- Systemic Immune-Inflammation Index (SII): (Neutrophils × Platelets) / Lymphocytes.
- Pan-Immune Inflammation Value (PIV): (Neutrophils × Platelets × Monocytes) / Lymphocytes.

Neutrophil-to-Lymphocyte Ratio (NLR) and Platelet-to-Lymphocyte Ratio (PLR).

### Statistical analysis

Statistical analyses were performed using R version 4.4.1. For continuous data with non‑normal distribution, group comparisons were conducted using the Kruskal–Wallis test and the Mann–Whitney U test, with results expressed as H and z statistics, respectively. The chi-squared test (χ² statistic) was used for preliminary screening of independent variables and for analysis of categorical data. Receiver operating characteristic (ROC) curve analysis was performed to evaluate the diagnostic value of each parameter in patients with AECOPD. Binary logistic regression analysis was applied to estimate the combined diagnostic probability of peripheral blood inflammatory markers; the corresponding ROC curves were constructed, and the area under the curve (AUC) was employed to compare the diagnostic performance of different modalities.

## Result

### Baseline characteristics of study population

A total of 148 participants were enrolled, including 49 healthy controls, 59 patients with AECOPD alone, and 40 patients with AECOPD complicated by PH. The baseline demographic and clinical characteristics are summarized in Table 1. Significant differences were observed across groups in age (H = 34.68, *p* < 0.001) and BMI (H = 6.69, *p* = 0.035). Post-hoc analysis revealed that both AECOPD and AECOPD + PH patients were significantly older than controls (both *p* < 0.001), while no significant age difference was detected between the two patient groups (*p* = 0.102). BMI was significantly lower in the AECOPD + PH group compared with controls (*p* = 0.011), but did not differ significantly between AECOPD and AECOPD + PH groups (*p* = 0.282).Sex distribution, smoking status, and history of hypertension were comparable among the three groups (all *p* > 0.05). Notably, the proportion of current smokers was higher in the AECOPD + PH group (85.0%) than in the AECOPD (64.4%) and control (61.2%) groups, although this difference did not reach statistical significance (*p* = 0.144).As expected, patients with AECOPD + PH had a significantly longer length of hospital stay than those with AECOPD alone (mean ± SD: 13.15 ± 6.74 vs. 6.48 ± 4.51 days; H = 496.5, *p* < 0.001).

These findings confirm that AECOPD patients with concomitant PH represent a more severe clinical phenotype characterized by advanced age, lower BMI, and prolonged hospitalization, despite comparable smoking and comorbidity profiles.

**Table 1:** Comparison of general information among control, AECOPD, and AECOPD + PH groups

General information	Control(49cases)	AECOPD(59cases)	AECOPD + PH(40cases)	Test statistic	*p*
Age	67.0 [47.0–76.0]	77.0 [73.0–80.0]	80.0 [73.5–87.0]	H = 34.68	< 0.001
Gender (male%)	31(63.3%)	37(62.7%)	31(77.5%)	*χ² =* 2.78	0.248
BMI(kg/m^2^)	22.38 [19.95–24.56]	20.40 [19.31–23.62]	20.50 [17.54–22.98]	*χ² =* 6.69	0.035
Smoking history(%)				*χ² =* 6.85	0.144
-never	11(22.4%)	8(16.3%)	30(61.2%)		
-former	12(20.3%)	9(15.3%)	38(64.4%)		
-current	4(10.0%)	2(5.0%)	34(85.0%)		
Hospitalization days	—	6.475 ± 4.512	13.15 ± 6.739	H = 496.5	< 0.001
Hypertension history	40.8%	49.2%	45.0%	*χ² =* 0.75	0.686

Abbreviations: AECOPD = Acute Exacerbation of COPD; PH = Pulmonary hypertension

### Inflammatory cytokine correlation analysis

**Fig. 1:**
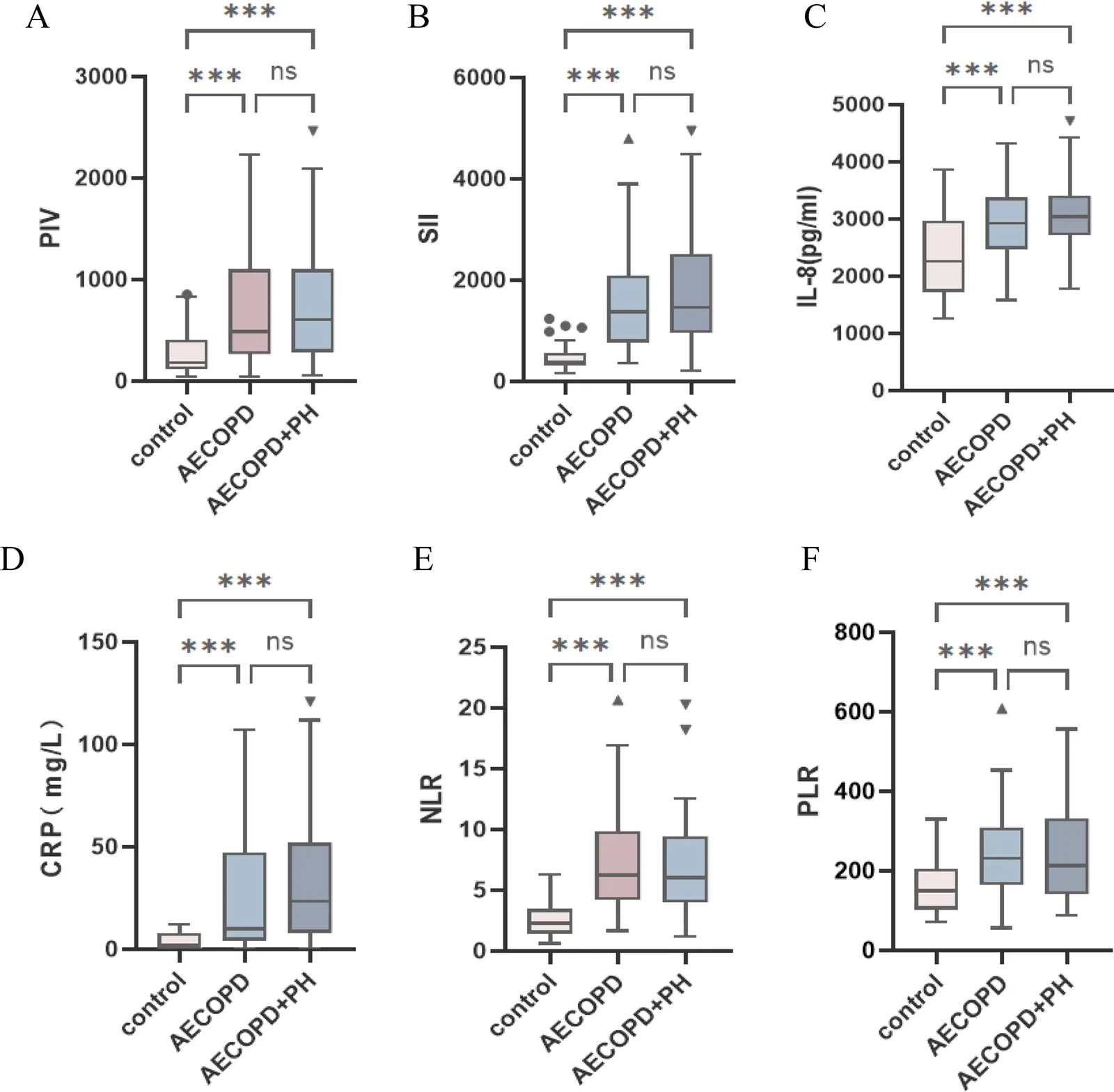
Comparison of inflammatory markers between control subjects and patients with AECOPD complicated by pulmonary hypertension (PH). Levels of systemic inflammatory and immune markers—including PIV (**A**), SII (**B**), IL-8 (**C**), CRP (**D**), NLR (**E**), and PLR (**F**)—were measured across groups. No significant differences were observed between the AECOPD and AECOPD + PH groups for any marker (ns, not significant). Data are presented as box plots; whiskers indicate minimum and maximum values, boxes represent the interquartile range, and the horizontal line denotes the median. ***p < 0.005. Abbreviations: PIV = (Neutrophil count × Monocyte count × Platelet count) / Lymphocyte count; SII = (Neutrophil count × Platelet count) / Lymphocyte count; NLR = Neutrophil count / Lymphocyte count; PLR = Platelet count / Lymphocyte count; AECOPD = Acute Exacerbation of COPD; PH = Pulmonary hypertension; PIV = Pan-Immune Inflammation Value; SII = Systemic Immune-Inflammation Index; NLR = Neutrophil-to-Lymphocyte Ratio; PLR = Platelet-to-Lymphocyte Ratio; CRP = C-reactive protein

Inflammatory cytokines serve as core biomarkers for assessing the severity of AECOPD, guiding treatment decisions, and predicting clinical outcomes. Therefore, relevant inflammatory markers including PIV, SII, IL-8, CRP, NLR, and PLR were measured. Based on comparisons using boxplot analyses according to quartiles, the study found that PIV, SII, IL-8, CRP, NLR, and PLR were all significantly up-regulated in both AECOD patients and those with AECOPD complicated by pulmonary hypertension compared to the control group. However, no statistically significant differences were observed in SII, IL-8, CRP, NLR, or PLR between AECOPD patients and those with AECOPD combined with pulmonary hypertension (Table S1; Fig. 1).

### Prothrombotic biomarker analysis

**Fig. 2:**
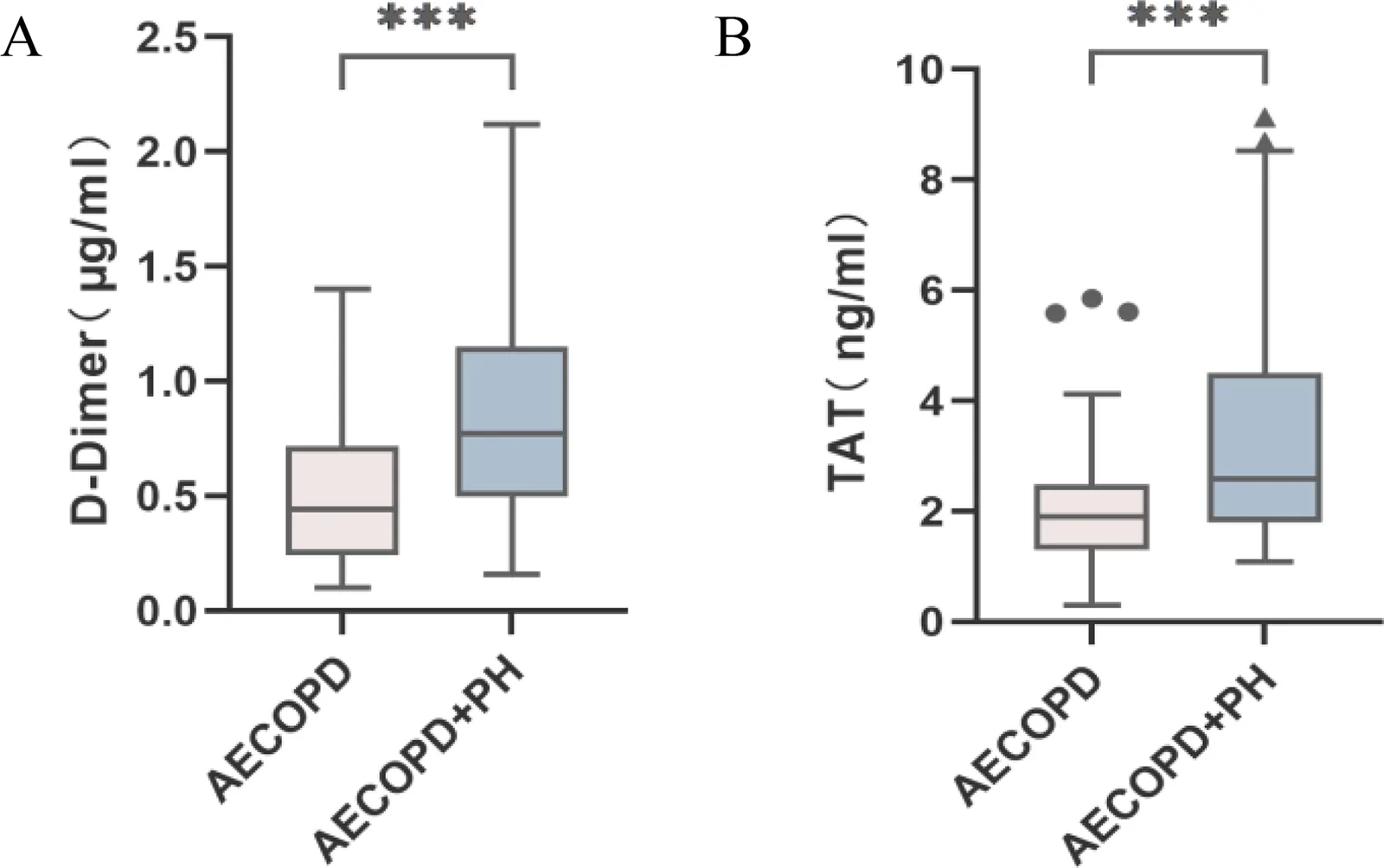
Comparison of coagulation biomarkers between AECOPD and AECOPD with PH groups. Plasma levels of thrombin-antithrombin complex (TAT) and D-dimer are significantly elevated in patients with AECOPD complicated by pulmonary hypertension compared to those with AECOPD alone. Data are presented as box and whiskers plots showing median, interquartile range, and minimum to maximum values. *p* < 0.05 between groups

In patients with AECOPD complicated by PH, vascular endothelial injury—driven by hypoxia and inflammation—leads to activation of the coagulation system. As shown in Fig. 2, comparative analysis of D-dimer and TAT levels between AECOPD patients and those with AECOPD plus PH revealed significantly higher measurements of both biomarkers in the AECOPD + PH group.

### Predictive performance of biomarkers for AECOPD and AECOPD with PH

**Fig. 3:**
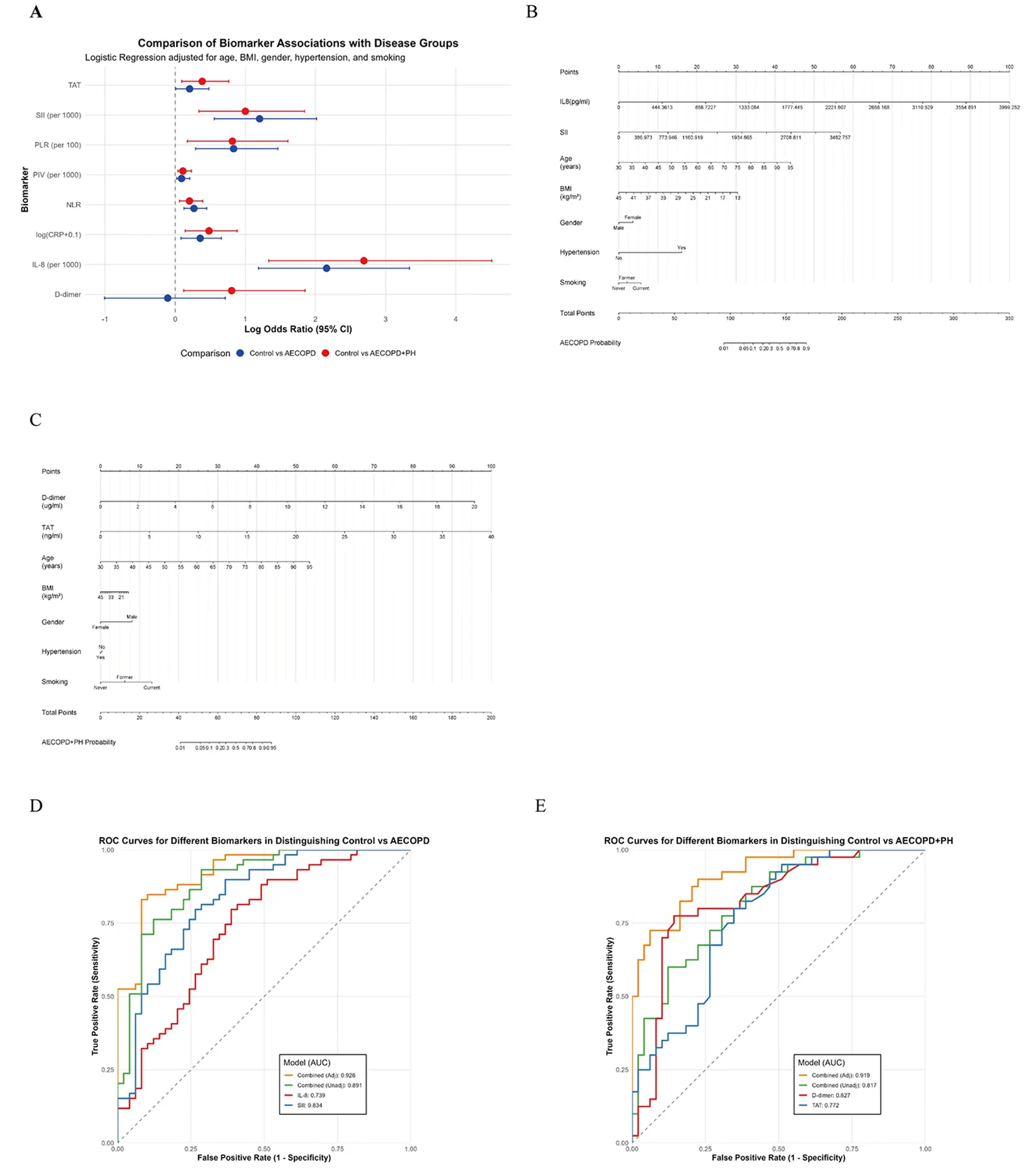
Predictive performance of biomarkers for AECOPD and AECOPD with PH (**A**) Forest plot displaying adjusted odds ratios (OR) and 95% confidence intervals (CI) for the association of each biomarker with disease status (Control vs. AECOPD, blue; Control vs. AECOPD + PH, red). Estimates were derived from logistic regression adjusted for age, BMI, gender, hypertension, and smoking status. (**B**) Nomogram for predicting the individual probability of AECOPD. The model includes IL-8 (pg/mL), SII, age (years), BMI (kg/m²), gender, hypertension, and smoking status (Never, Current). Each predictor is assigned points according to its value; the total points (0-350) correspond to the predicted AECOPD probability (0.01–0.80). (**C**) Nomogram for predicting the individual probability of pulmonary hypertension in patients with AECOPD (AECOPD + PH). The model incorporates D-dimer (µg/mL), TAT (ng/mL), age (years), BMI (kg/m²), gender, hypertension, and smoking status (Never, Current). Total points (0-200) translate directly into the predicted AECOPD + PH probability (0.01–0.965). (**D**) Receiver operating characteristic (ROC) curves comparing the discriminative performance of IL-8, SII, their unadjusted combination, and the covariate-adjusted combination for distinguishing AECOPD patients from healthy controls (Control vs. AECOPD). The adjusted model includes age, BMI, gender, hypertension, and smoking status. Area under the curve (AUC) values are shown in the panel. (**E**) ROC curves evaluating the ability of D-dimer, TAT, their unadjusted combination, and the covariate-adjusted combination for distinguishing AECOPD patients with pulmonary hypertension from healthy controls (Control vs. AECOPD + PH). AUC values are presented within the panel

To identify independent predictors and develop clinically applicable tools, multivariable logistic regression models were constructed with adjustment for age, BMI, gender, hypertension, and smoking status. Figure 3A and supplement Table 2 illustrates the adjusted associations of candidate biomarkers with the presence of AECOPD and AECOPD + PH relative to healthy controls. Compared with controls, both AECOPD and AECOPD + PH groups demonstrated significantly elevated odds ratios for IL-8, SII, NLR, PLR, and log (CRP + 0.1) (all *p* < 0.05) Among them, the increases in IL-8 and SII were more significant. In contrast, D-dimer and TAT exhibited stronger and more selective associations with the AECOPD + PH group.

Based on the final regression coefficients, two prediction nomograms were constructed. Figure 3B shows the nomogram for estimating the individual probability of AECOPD. Higher levels of IL‑8 and SII, older age, lower BMI, male sex, presence of hypertension, and current smoking contributed more points, leading to a higher predicted risk of AECOPD. Figure 3C presents the nomogram for estimating the probability of PH among AECOPD patients. Elevated D-dimer and TAT, older age, lower BMI, male sex, absence of hypertension, and current smoking were associated with increased total points and thus a greater predicted probability of AECOPD + PH.

ROC curve analyses were performed to evaluate the discriminative ability of individual biomarkers and their combinations (Table S3). For distinguishing AECOPD patients from healthy controls (Fig. 3D), IL-8 alone yielded an AUC of 0.739 (95% CI: 0.642–0.832), SII alone an AUC of 0.834 (95% CI: 0.748–0.905). After full adjustment for covariates, the combined model achieved the highest AUC of 0.926 (95% CI: 0.876–0.967).For distinguishing AECOPD patients with PH from healthy controls (Fig. 3E), D-dimer alone exhibited an AUC of 0.827 (95% CI: 0.735–0.918), TAT alone an AUC of 0.772 (95% CI: 0.667–0.855).The covariate-adjusted combined model demonstrated superior discriminative performance, with an AUC of 0.919 (95% CI: 0.865–0.966).

Collectively, these results demonstrate that systemic inflammatory markers (IL-8, SII) are robust predictors of AECOPD, whereas prothrombotic markers (D-dimer, TAT) are more specifically associated with the presence of PH in the setting of AECOPD. The constructed nomograms and combined ROC models provide quantitative, individualized risk assessment tools that may facilitate early identification of high-risk patients.

### Mechanism diagram

**Fig. 4:**
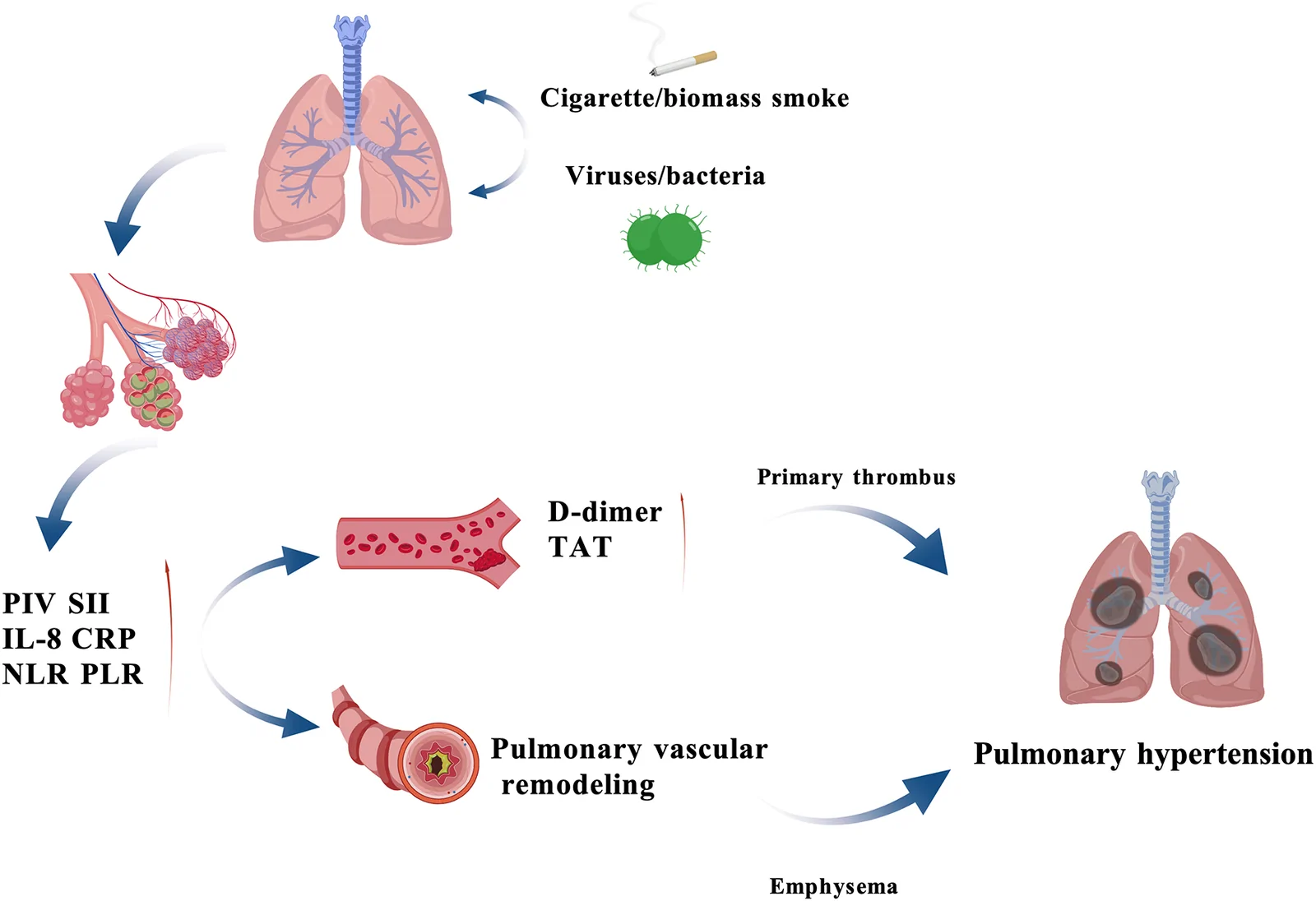
Proposed Pathophysiological Mechanism Linking AECOPD and Pulmonary Hypertension (PH). This schematic illustrates that during AECOPD, levels of pro-inflammatory cytokines are significantly elevated. In AECOPD patients who develop concomitant PH, this is associated with a further rise in prethrombotic markers, indicating a pronounced hypercoagulable state

As delineated in Fig. 4, the acute exacerbation phase of COPD (AECOPD) is characterized by a substantial upregulation of systemic inflammatory mediators. Furthermore, the subset of AECOPD patients complicated by pulmonary hypertension (PH) demonstrates a marked additional increase in circulating prethrombotic biomarkers, suggesting an enhanced prothrombotic milieu.

## Discussion

This study delineates a divergent pathophysiological trajectory in AECOPD patients with concomitant PH: systemic inflammation reaches an elevated plateau, whereas coagulation activation intensifies progressively.This divergent trajectory is supported by three core findings.

First, circulating inflammatory markers (PIV, SII, IL-8, CRP, NLR, PLR) were uniformly elevated in both AECOPD and AECOPD + PH groups versus controls, yet no further escalation accompanied the development of PH.Despite marked elevations relative to healthy controls, the absence of a further inflammatory surge in AECOPD-PH patients contrasts partially with prior reports [32] observed higher NLR, PLR, and SII in AECOPD-PH versus AECOPD alone; however, multivariable adjustment abrogated these associations, it may suggest residual confounding by disease severity or comorbidities.The discrepancy may stem from differences in cohort characteristics our patients were older, had longer disease duration, and higher smoking prevalence factors that promote chronic low-grade inflammation with blunted acute responsiveness, a phenotype compatible with immune exhaustion [33]. Moreover, inflammatory trajectories are biomarker-specific; progressive elevation of CA125 [34] in AECOPD-PH highlights heterogeneity across mediators.Furthermore, the prognostic value of these inflammatory markers for other AECOPD-related complications has been documented in several studies [35] demonstrated that CRP predicts 90-day readmission more robustly than sputum inflammatory markers [36] identified NLR, PLR, and SII as independent predictors of frequent exacerbations [21] recently showed that the same biomarkers—NLR, PLR, SII—independently predict symptomatic venous thromboembolism in hospitalized AECOPD patients. Collectively, these studies affirm that systemic inflammation drives multiple adverse outcomes in AECOPD. Our findings suggest that PH may represent an exception to the paradigm of inflammation-driven AECOPD complications: despite its established association with inflammatory processes, the development of PH in our cohort was not accompanied by a parallel systemic inflammatory surge.We hypothesize that this phenomenon may reflect an inflammatory plateau or systemic immune exhaustion, and suggest that AECOPD-PH could represent a distinct endotype diverging from other inflammatory-driven comorbidities.

Second, in marked contrast to the static inflammatory milieu, D-dimer and TAT exhibited a stepwise increase from AECOPD alone to AECOPD + PH. Multivariable analysis confirmed both as independent predictors of PH (D-dimer: aOR = 2.17 per µg/mL; TAT: aOR = 1.50 per µg/L). This progressive coagulopathy is consistent with the established role of coagulation markers in AECOPD. incorporated D-dimer into a predictive model for symptomatic VTE, underscoring its thrombotic risk association. The present findings suggest that PH may confer an additional prothrombotic risk in patients with AECOPD-an observation that, to our knowledge, has not been previously reported in the literature. The coexistence of an inflammatory plateau and progressive coagulopathy may point to a distinct biomarker phenomenon, potentially offering a novel rationale for anticoagulation in selected patients with AECOPD-PH.

Third, ROC analyses quantified diagnostic utility. For AECOPD detection, SII (AUC = 0.834) and IL-8 (AUC = 0.739) individually, and their covariate-adjusted combination (AUC = 0.926), exhibited excellent performance. For PH identification among AECOPD patients, the adjusted D-dimer + TAT model achieved an AUC of 0.919, substantially outperforming single markers.

We speculate that the observed inflammatory plateau may reflect either localized pulmonary vascular inflammation, which may evade accurate detection in peripheral blood, or systemic immune exhaustion, a concept that remains speculative and requires further investigation in this context.The concept of immune exhaustion, originally described in the context of chronic infection and malignancy, has been characterized by diminished effector cytokine production and attenuated responsiveness to persistent antigenic stimulation. Whether a analogous phenomenon occurs in the setting of recurrent AECOPD and contributes to blunted systemic inflammatory responses in patients with PH remains speculative and warrants further investigation. Conversely, hemodynamic alterations secondary to PH may promote sustained activation of the coagulation cascade. This dissociation between quiescent systemic inflammation and progressive hypercoagulability may represent a distinct pathophysiological phenotype of AECOPD‑PH.

Our study is subject to several limitations. First, due to its cross-sectional, case-control design, causality between observed biomarker profiles and disease status cannot be inferred; longitudinal studies are required to delineate temporal dynamics. Second, the single-center, retrospective nature and relatively modest sample size constrain generalizability. Although a post-hoc power analysis confirmed adequate statistical power (> 90%) for detecting our primary findings (e.g.differences in prothrombotic markers), the study may have been underpowered to identify more subtle distinctions, such as in systemic inflammatory markers between AECOPD and AECOPD + PH groups, necessitating cautious interpretation of these null results. Finally, the diagnosis of pulmonary hypertension relied solely on echocardiography rather than the gold-standard right heart catheterization, which may introduce classification bias. These limitations underscore the need for larger, prospective, multi-center studies incorporating invasive hemodynamic confirmation to validate and extend our observations.

Therefore, the primary contribution of this study is not the isolated reporting of inflammation or hypercoagulability, but the integrated observation of their dissimilar evolution in AECOPD-PH. The pattern—an early plateau in systemic inflammation concurrent with a rising trajectory of coagulation activation—provides a new pathophysiological framework. It challenges the assumption of a simple additive effect and substantiates our proposed hypotheses that either a compartmentalized pulmonary vascular inflammatory focus or a state of systemic immune exhaustion may characterize the progression to PH in AECOPD. This dissociation between inflammatory and coagulation biomarker dynamics underscores AECOPD-PH as a distinct endotype with potential implications for targeted management strategies.

## Supplementary Information

Below is the link to the electronic supplementary material.

Supplementary Material 1

## Data Availability

No datasets were generated or analysed during the current study.

## Abbreviations

AECOPD: Acute Exacerbation of Chronic Obstructive Pulmonary Disease
CRP: C-reactive protein
IL-8: Interleukin-8
NLR: Neutrophil-to-Lymphocyte Ratio
PASP: Pulmonary artery systolic pressure
PCT: Procalcitonin
PH: Pulmonary Hypertension
PIC: Plasmin-α2-plasmin inhibitor complex
PIV: Pan-immune-inflammation value
PLR: Platelet-to-Lymphocyte Ratio
PTS: Prothrombotic state
RHC: Right heart catheterization
ROC: Receiver operating characteristic
SII: Systemic Immune-Inflammation Index
TAT: Thrombin-antithrombin complex
TTE: Transthoracic echocardiography
t-PAIC: Tissue plasminogen activator-inhibitor complex

## References

[1] Global regional. national age-sex-specific mortality for 282 causes of death in 195 countries and territories, 1980–2017: a systematic analysis for the Global Burden of Disease Study 2017 [J]. Lancet. 2018;392(10159):1736–88. 10.1016/S0140-6736(18)32203-7 PMC6227606 30496103

[2] Wang C, Xu J, Yang L, et al. Prevalence and risk factors of chronic obstructive pulmonary disease in China (the China Pulmonary Health [CPH] study): a national cross-sectional study [J]. Lancet. 2018;391(10131):1706–17. 10.1016/S0140-6736(18)30841-9 29650248

[3] Vestbo J, Hurd SS, Agustí AG, et al. Global strategy for the diagnosis, management, and prevention of chronic obstructive pulmonary disease: GOLD executive summary [J]. Am J Respir Crit Care Med. 2013;187(4):347–65. 10.1164/rccm.201204-0596PP 22878278

[4] Halpin DMG, Celli BR, Criner GJ, et al. The GOLD Summit on chronic obstructive pulmonary disease in low- and middle-income countries [J]. Int J Tuberc Lung Dis. 2019;23(11):1131–41. 10.5588/ijtld.19.0397 31718748

[5] Singh R, Mackay AJ, Patel AR, et al. Inflammatory thresholds and the species-specific effects of colonising bacteria in stable chronic obstructive pulmonary disease [J]. Respir Res. 2014;15(1):114. 10.1186/s12931-014-0114-1 PMC4173051 25218165

[6] Gredic M, Blanco I, Kovacs G, et al. Pulmonary hypertension in chronic obstructive pulmonary disease [J]. Br J Pharmacol. 2021;178(1):132–51. 10.1111/bph.14979 31976545

[7] Hoogendoorn M, Hoogenveen RT, Rutten-van Mölken MP, et al. Case fatality of COPD exacerbations: a meta-analysis and statistical modelling approach [J]. Eur Respir J. 2011;37(3):508–15. 10.1183/09031936.00043710 20595157

[8] Ko FW, Chan KP, Hui DS, et al. Acute exacerbation of COPD [J]. Respirology. 2016;21(7):1152–65. 10.1111/resp.12780 PMC7169165 27028990

[9] Yuanxu Dong JZ, Feiyanwei Y, Wei. Exploration of the timing of invasive mechanical ventilation treatment and analysis of prognostic factors in patients with AECOPD and severe respiratory failure [J]. Progress Mod Biomedicin. 2023;23:09.

[10] Wang L, Wu Y, Jiang X, Yan W, Wang J. Observation of the clinical efficacy of milinong combined with danshen polyphenolic acid salt in the treatment of AECOPD with pulmonary hypertension [J]. New Medicine. 2015;46:12.

[11] Çolak Y, Afzal S, Lange P, Nordestgaard BG, Smoking. Systemic Inflammation, and Airflow Limitation: A Mendelian Randomization Analysis of 98 085 Individuals From the General Population [J]. Nicotine Tob Res. 2019;21(8):1036–44. 10.1093/ntr/nty077 29688528

[12] Strollo HC, Nouraie SM, Hoth KF, et al. Association of Systemic Inflammation with Depressive Symptoms in Individuals with COPD [J]. Int J Chron Obstruct Pulmon Dis. 2021;16:2515–22. 10.2147/COPD.S322144 PMC8423410 34511896

[13] Su G. The effect of clopidogrel on hypercoagulability and lung function in patients with acute exacerbation of chronic obstructive pulmonary disease [J]. Int J Geriatric Med. 2019;40(2):3.

[14] Ugurlu E, Kilic-Toprak E, Can I, et al. Impaired hemorheology in exacerbations of COPD [J]. Can Respir J. 2017;2017:1286263. 10.1155/2017/1286263 PMC5635272 29089816

[15] Shi X, Li H. Anticoagulation therapy in patients with chronic obstructive pulmonary disease in the acute exacerbation stage [J]. Exp Ther Med. 2013;5(5):1367–70. 10.3892/etm.2013.1001 PMC3671742 23737881

[16] Sabit R, Thomas P, Shale DJ, et al. The effects of hypoxia on markers of coagulation and systemic inflammation in patients with COPD [J]. Chest. 2010;138(1):47–51. 10.1378/chest.09-2764 20154074

[17] Børvik T, Brækkan SK, Enga K, et al. COPD and risk of venous thromboembolism and mortality in a general population [J]. Eur Respir J. 2016;47(2):473–81. 10.1183/13993003.00402-2015 26585434

[18] Bai PCD, Sun YC. Relationship between CRP, D-dimer and pulmonary hypertension in patients with AECOPD [J]. China J Mod Med. 2009;19(8):1204–7.

[19] Hu B, Yang XR, Xu Y, et al. Systemic immune-inflammation index predicts prognosis of patients after curative resection for hepatocellular carcinoma [J]. Clin Cancer Res. 2014;20(23):6212–22. 10.1158/1078-0432.CCR-14-0442 25271081

[20] Kerr R, Stirling D, Ludlam CA. Interleukin 6 and haemostasis [J]. Br J Haematol. 2001;115(1):3–12. 10.1046/j.1365-2141.2001.03061.x 11722403

[21] Zeng J, Feng J, Luo Y, et al. Inflammatory Biomarkers as Predictors of Symptomatic Venous Thromboembolism in Hospitalized Patients with AECOPD: A Multicenter Cohort Study [J]. J Atheroscler Thromb. 2025;32(4):439–57. 10.5551/jat.65177 PMC11973524 39477518

[22] Saghazadeh A, Hafizi S, Rezaei N. Inflammation in venous thromboembolism: Cause or consequence? [J]. Int Immunopharmacol. 2015;28(1):655–65. 10.1016/j.intimp.2015.07.044 26253657

[23] Tillie-Leblond I, Marquette CH, Perez T, et al. Pulmonary embolism in patients with unexplained exacerbation of chronic obstructive pulmonary disease: prevalence and risk factors [J]. Ann Intern Med. 2006;144(6):390–6. 10.7326/0003-4819-144-6-200603210-00005 16549851

[24] Delluc A, Tromeur C, Le Ven F, et al. Current incidence of venous thromboembolism and comparison with 1998: a community-based study in Western France [J]. Thromb Haemost. 2016;116(5):967–74. 10.1160/TH16-03-0205 27465905

[25] Tapson VF. The role of smoking in coagulation and thromboembolism in chronic obstructive pulmonary disease [J]. Proc Am Thorac Soc. 2005;2(1):71–7. 10.1513/pats.200407-038MS 16113472

[26] Gamble E, Grootendorst DC, Hattotuwa K, et al. Airway mucosal inflammation in COPD is similar in smokers and ex-smokers: a pooled analysis [J]. Eur Respir J. 2007;30(3):467–71. 10.1183/09031936.00013006 17504799

[27] Group C, O P D M w I T C a W M E C W, Quanying H, Cuiling F. Expert Consensus on Integrated Traditional Chinese and Western Medicine Management of Chronic Obstructive Pulmonary Disease (2023 Edition) [J]. Chin Gen Pract J. 2023;26(35):4359–71.

[28] Adeloye D, Song P, Zhu Y, et al. Global, regional, and national prevalence of, and risk factors for, chronic obstructive pulmonary disease (COPD) in 2019: a systematic review and modelling analysis [J]. Lancet Respir Med. 2022;10(5):447–58. 10.1016/S2213-2600(21)00511-7 PMC9050565 35279265

[29] Garcia JJ, Herranz LS, García SG, et al. Concordance among GOLD, NICE and LLN criteria in the spirometry diagnosis of airflow obstruction [J]. Eur Respir J. 2016;48(suppl 60):PA4625.

[30] (CMA) C M A, House C M A P, Association GP, B o t C M, et al. Chinese Guideline for Primary Care Diagnosis, Treatment and Management of Chronic Obstructive Pulmonary Disease (2024) [J]. Chin J Gen Practitioners. 2024;23(06):578–602.

[31] Sood A, Petersen H, Qualls C, et al. Spirometric variability in smokers: transitions in COPD diagnosis in a five-year longitudinal study [J]. Respir Res. 2016;17(1). 10.1186/s12931-016-0468-7 PMC5105293 27832774

[32] Zuo H, Xie X, Peng J, et al. Predictive value of novel inflammation-based biomarkers for pulmonary hypertension in the acute exacerbation of chronic obstructive pulmonary disease [J]. Anal Cell Pathol (Amst). 2019;2019:5189165. 10.1155/2019/5189165 PMC6815641 31737467

[33] Baessler A, Vignali DAA. T Cell Exhaustion [J]. Annu Rev Immunol. 2024;42(1):179–206. 10.1146/annurev-immunol-090222-110914 38166256

[34] Li S, Ma H, Gan L, et al. Cancer antigen-125 levels correlate with pleural effusions and COPD-related complications in people living at high altitude [J]. Med (Baltim). 2018;97(46):e12993. 10.1097/MD.0000000000012993 PMC6257551 30431573

[35] Jing Z, Chun C, Ning S, et al. Systemic Inflammatory Marker CRP Was Better Predictor of Readmission for AECOPD Than Sputum Inflammatory Markers [J]. Arch Bronconeumol. 2016;52(3):138–44. 10.1016/j.arbres.2015.01.011 26002550

[36] Fu Y, Wang Y, Wang Y, et al. Biomarkers (NLR, PLR, SII) for Frequent COPD Exacerbations: Diagnostic and Clinical Management Implications in a Retrospective Study [J]. Int J Chron Obstruct Pulmon Dis. 2025;20:987–98. 10.2147/COPD.S510118 PMC11980941 40207023

