# Supplementary material for: Differences in systemic inflammation and prethrombotic biomarkers between AECOPD patients with and without pulmonary hypertension
Source: J Inflamm (Lond). 2026 Jul 15;23:26. doi: 10.1186/s12950-026-00508-7 (PMC13651094; doi:10.1186/s12950-026-00508-7)
Supplement: Supplementary file 1 [file 12950_2026_508_MOESM1_ESM.docx]

**Table S1. Univariate Logistic Regression Analysis of Biomarkers for Predicting AECOPD and AECOPD+PH (Reference: Control Group)**

| Biomarker | Control vs AECOPD | | Control vs AECOPD+PH | |
| --- | --- | --- | --- | --- |
|  | 95% CI | P value | 95% CI | P value |
| IL-8 (per 1000 pg/mL) | 8.65 [3.28]-[28.24] | <0.001 | 14.73 [3.79]-[91.35] | <0.001 |
| log(CRP+0.1) (mg/L) | 1.43 [1.09]-[1.93] | 0.014 | 1.62 [1.15]-[2.42] | 0.009 |
| NLR (ratio) | 1.31 [1.13]-[1.57] | 0.001 | 1.23 [1.06]-[1.48] | 0.015 |
| D-dimer (μg/mL) | 0.90 [0.37]-[2.04] | 0.786 | 2.24 [1.13]-[6.37] | 0.058 |
| TAT (μg/L) | 1.23 [1.01]-[1.62] | 0.083 | 1.47 [1.10]-[2.15] | 0.022 |
| PIV (per 1000) | 1.09 [1.02]-[1.23] | 0.055 | 1.12 [1.04]-[1.26] | 0.023 |
| SII (per 1000) | 3.33 [1.75]-[7.50] | 0.001 | 2.72 [1.40]-[6.33] | 0.008 |
| PLR (per 100) | 2.30 [1.34]-[4.33] | 0.005 | 2.26 [1.19]-[4.99] | 0.024 |

#### Table S2. Multivariable Logistic Regression Analysis for Different Clinical Groups

| Predictor | β | 95% CI | Wald Z | P value |
| --- | --- | --- | --- | --- |
| AECOPD vs Control | | | | |
| IL-8 (per 1000 pg/mL) | 2.2985 | 9.96 [3.61]-[35.4] | 4 | <0.001 |
| SII (per 1000) | 1.4968 | 4.47 [2.03]-[12.1] | 3.34 | <0.001 |
| Age (per year) | 0.0621 | 1.06 [1.02]-[1.13] | 2.42 | 0.015 |
| BMI (per kg/m²) | -0.0875 | 0.92 [0.77]-[1.05] | -1.10 | 0.271 |
| Gender (Female vs. Male) | 0.3336 | 1.40 [0.41]-[5.06] | 0.53 | 0.599 |
| Hypertension (Yes vs. No) | 1.4833 | 4.41 [1.17]-[19.7] | 2.09 | 0.037 |
| Smoking (Former vs. Never) | 0.1949 | 1.22 [0.16]-[9.24] | 0.19 | 0.848 |
| Smoking (Current vs. Never) | 0.524 | 1.69 [0.36]-[8.39] | 0.66 | 0.506 |
| AECOPD+PH vs AECOPD | | | | |
| D- dimer (per μg/mL) | 0.7746 | 2.17 [1.11]-[5.76] | 1.99 | 0.046 |
| TAT (per μg/L) | 0.4046 | 1.50 [1.09]-[2.27] | 2.21 | 0.027 |
| Age (per year) | 0.1333 | 1.14 [1.07]-[1.26] | 3.15 | 0.002 |
| BMI (per kg/m²) | -0.0360 | 0.96 [0.83]-[1.09] | -0.55 | 0.586 |
| Gender (Female vs. Male) | -1.3051 | 0.27 [0.05]-[1.15] | -1.70 | 0.089 |
| Hypertension (Yes vs. No) | -0.0623 | 0.94 [0.23]-[3.79] | -0.09 | 0.929 |
| Smoking (Former vs. Never) | 1.0076 | 2.74 [0.09]-[53.1] | 0.66 | 0.51 |
| Smoking (Current vs. Never) | 2.1262 | 8.38 [1.29]-[74.6] | 2. 1 | 0.036 |

**Table S3. Comprehensive ROC Analysis for Discriminating AECOPD from Controls**

| Model | AUC | Sensitivity | Specificity | Accuracy | Youden_Index |
| --- | --- | --- | --- | --- | --- |
| IL-8 (per 1000 pg/mL) | 0.739 | 0.797 | 0.612 | 0.713 | 0.409 |
| SII (per 1000) | 0.834 | 0.797 | 0.735 | 0.769 | 0.533 |
| IL-8+SII Combined Score (Unadjusted) | 0.891 | 0.932 | 0.714 | 0.833 | 0.646 |
| IL-8+SII Combined Score (Adjusted) | 0.926 | 0.831 | 0.918 | 0.870 | 0.749 |
| D-dimer (per μg/mL) | 0.827 | 0.775 | 0.857 | 0.820 | 0.632 |
| TAT (per μg/L) | 0.772 | 0.800 | 0.653 | 0.719 | 0.453 |
| D-dimer+TAT Combined Score (Unadjusted) | 0.817 | 0.600 | 0.878 | 0.753 | 0.478 |
| D-dimer+TAT Combined Score (Adjusted) | 0.919 | 0.900 | 0.776 | 0.831 | 0.676 |
